# Highlight: Warning Signs in a Poisonous Papuan Songbird

**DOI:** 10.1093/gbe/evz169

**Published:** 2019-08-24

**Authors:** Casey McGrath

Bright colors and conspicuous markings are often used in nature to warn off would-be predators. Although we are used to seeing such markings—termed aposematic signals—in plants, caterpillars, and snakes, we do not usually think of colorful bird plumage as conveying the same message. However, members of the New Guinea songbird genus *Pitohui* use their plumage to warn predators that they are toxic.

Aposematic coloration often gives rise to so-called Müllerian mimicry rings, in which multiple toxic species evolve to resemble each other, as a mutual form of protection. The theory is that predators will more quickly learn to avoid all of the species in the ring, with less of a cost to each species. Although these rings are common in butterflies and other insects, they are less common among vertebrates where the genetic basis of these traits is often unknown. This is certainly true for pitohuis, one of the few toxic birds on Earth. In a new article in *Genome Biology and Evolution*, “Gene flow in the Müllerian mimicry ring of a poisonous Papuan songbird clade (*Pitohui*; Aves)” (Garg et al. 2019), an international team of researchers from the National University of Singapore, Biology Centre CAS in the Czech Republic, and the New Guinea Binatang Research Centre set out to unravel the genetic basis and evolutionary history of pitohuis’ colorful plumage.

There are several possible explanations for the evolution of Müllerian mimicry. Early hypotheses suggested that species involved in the ring converged on similar markings through different genetic mechanisms. In pitohuis, it has also been proposed that aposematic coloration is an ancestral trait. In butterflies, introgression, which involves the introduction of genetic material from one species to another through interbreeding, has been suggested to play a role. In their study, the first author Dr Kritika Garg and colleagues investigated these possibilities in three pitohui species: the hooded pitohui (*Pitohuidichrous*), which has highly aposematic coloring and is toxic across its entire range, and the northern variable pitohui (*Pitohuikirhocephalus*) and southern variable pitohui (*Pitohuiuropygialis*), both of which vary in coloration and toxicity across their ranges (fig. 1).

**Fig. 1.**
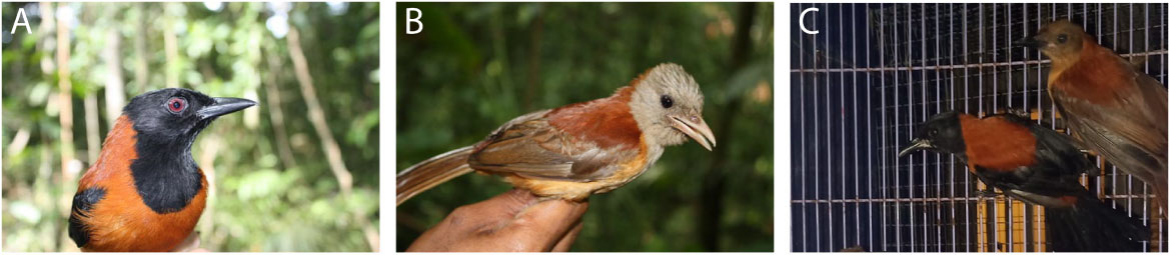
—(*A*) Hooded pitohui, (*B*) northern variable pitohui, and (*C*) southern variable pitohui (left). Images provided by Katerina Sam (*A* and *B*) and Carlos Gonzalez Bocos (*C*).

Because gene flow between species can blur evolutionary trajectories, the authors used several genomic approaches to determine the relationships between the three species and the evolutionary history of pitohui coloration. Using genome-wide markers from dozens of birds, the authors’ results provide clear evidence for introgression from the hooded pitohui to the southern variable pitohui. Moreover, regions of the genome derived from the hooded pitohui were linked to genes potentially involved in plumage coloration and toxin resistance. Together, these findings suggest that hooded pitohuis occasionally mate with southern variable pitohuis and, in the process, share alleles involved in coloration and toxin resistance.

More broadly, these data contribute to a fundamental reassessment of how Müllerian mimicry rings work. According to corresponding author Frank Rheindt, “Independent evolution of mimetic traits has remained the explanation of choice for most vertebrate mimicry rings. By demonstrating introgression among mimetic songbirds, our paper strongly suggests that gene flow should henceforth be considered the null hypothesis for generating Müllerian mimicry rings.” However, the authors also point out that, “While our work identified [genes] implicated in toxicity resistance and plumage coloration that are potentially transferred among mimetic species by gene flow, a firm functional link has yet to be established.”

The researchers hope to further explore the link between genotype and phenotype in pitohuis and to characterize the functional underpinnings of plumage coloration and toxicity in future work. According to Dr Rheindt, “Our interest in pitohuis goes back many years and is grounded in arduous field research in the Papuan rainforests during which we have had firsthand experience of observing this mimicry ring.” However, field operations chief Dr Katerina Sam, who was wholly responsible for collecting the material used in this study, notes that “Fieldwork in Papua continues to be extremely laborious and challenging.” Thus, while the authors’ sampling regime for this study was as comprehensive as conditions allowed, they are excited to expand on the current work in future endeavors.

## References

[evz169-B1] Garg KM, et al Forthcoming 2019 Gene flow in the Müllerian mimicry ring of a poisonous Papuan songbird clade (*Pitohui*; Aves). Genome Biol Evol. 10.1093/gbe/evz168 PMC6735254 31418795

